# Plant Development and Crop Yield: The Role of Gibberellins

**DOI:** 10.3390/plants11192650

**Published:** 2022-10-09

**Authors:** Ricardo Castro-Camba, Conchi Sánchez, Nieves Vidal, Jesús Mª Vielba

**Affiliations:** Misión Biológica de Galicia, Consejo Superior de Investigaciones Científicas, 15780 Santiago de Compostela, Spain

**Keywords:** agricultural applications, biotechnology, gene regulation, gibberellins, phytohormones, plant growth

## Abstract

Gibberellins have been classically related to a few key developmental processes, thus being essential for the accurate unfolding of plant genetic programs. After more than a century of research, over one hundred different gibberellins have been described. There is a continuously increasing interest in gibberellins research because of their relevant role in the so-called "Green Revolution", as well as their current and possible applications in crop improvement. The functions attributed to gibberellins have been traditionally restricted to the regulation of plant stature, seed germination, and flowering. Nonetheless, research in the last years has shown that these functions extend to many other relevant processes. In this review, the current knowledge on gibberellins homeostasis and mode of action is briefly outlined, while specific attention is focused on the many different responses in which gibberellins take part. Thus, those genes and proteins identified as being involved in the regulation of gibberellin responses in model and non-model species are highlighted. The present review aims to provide a comprehensive picture of the state-of-the-art perception of gibberellins molecular biology and its effects on plant development. This picture might be helpful to enhance our current understanding of gibberellins biology and provide the know-how for the development of more accurate research and breeding programs.

## 1. Introduction

Phytohormones are a chemically diverse set of compounds that regulate plant development at micromolar concentrations. Hormone synthesis, transport, and degradation are tightly controlled because minor variations of their levels in tissues can have a huge impact on plant responses as they play important roles in the regulation of gene expression or the activity of other hormones.

Gibberellins (GAs) can be included as one of the five classical hormones, along with auxins, cytokinins, abscisic acid, and ethylene [1]. Each of these groups of hormones is associated with specific plant traits and physiological responses. In the case of GAs, they have been usually associated with the regulation of plant stature [2] and seed dormancy [3]. However, results in recent years have shown that this might be an oversimplification, and GAs (as well as the rest of phytohormones) have direct or indirect effects on the regulation of many plant traits. GAs were key elements in the Green Revolution that took place within the second half of the 20th century, and many of the plant varieties with improved agronomical traits (dwarf phenotypes, increased biomass) showed to be related to GA activity and signaling [4]. However, the potential innovation of GA is far from exhausted and they can be again the leader of a new Green revolution [5] increasing yield and improving nitrogen-use efficiency all at once [6].

In the present review, an update on the recent findings concerning the many facets of development in which GA take part is intended. Besides, we also focus on the current and potential applications of GA in crop production, highlighting the relevance of these compounds as regulatory agents in agriculture. The emphasis is on the distinct effects of these hormones in plant responses through the modulation of gene expression and the agronomical impact of GA and related compounds. Those genes and proteins identified within the GA signaling cascades are underlined due to their potential interest as targets for future breeding programs. By gathering information from different species, our aim is to integrate molecular data that might help in the development of conceptual models regarding GA activity. However, as it will be shown, the variability in the responses in different species, the diversity of GA-related compounds, and the lack of specific research in some fields hinder the development of such models except for particular processes. Nonetheless, we hope that this review might be useful for researchers and growers in the definition of their strategies.

## 2. History of Gibberellins Research

GAs are a type of phytohormones first uncovered in the early 20th century [7] during the study of a common rice disease known as *bakanae*, which causes significant losses every year [8]. The causing agent of this condition is the fungus *Fusarium fujikuroi*, an ascomycete that spreads through water and infects the seeds. The infected plants are characterized by a very thin and elongated stem, resulting in a somehow ridiculous ("foolish") aspect. An over-accumulation of GA produced by the fungus during seed infection is the reason underlying this phenotype, which also concurs with etiolation and infertility [9]. During the 1950s, efforts conducted by Japanese scientists led to the isolation of gibberellin A_1_, gibberellin A_2_, gibberellin A_3_ (later known as gibberellic acid), and gibberellin A_4_ [10,11]. Within the same period, the normal phenotype of maize dwarf mutants was restored after GA_3_ application [12]. Further investigation guided the characterization of new GAs in plants, fungi, and bacteria (reviewed in [13]).

GAs have had a huge relevance due to their direct impact on agricultural performance. The characterization of dwarf varieties of wheat and rice showed the implication of GA in the resulting phenotypes of these plants. This short stature turned out to be an interesting agronomic trait for several crops as it reduced the risk of lodging, while providing more compact ornamentals [14]. In this way, GA modulation played a key role in the Green Revolution because of the increase in grain yield, harvest index of these varieties, and improved stress resistance to wind and rain due to their compactness [15]. A deeper comprehension of GA molecular activity and its application to accurate breeding programs may help to ease the path for new advances in crop management and a better and more sustainable food production [16].

Fundamental molecular aspects of GA synthesis, homeostasis, and signaling have been described in the last years, although several issues concerning transport or interactions with other phytohormones are still not fully elucidated [17]. GAs are acid diterpenoids derived from the terpenes route. The amount of bioactive GAs in plant tissues is determined by the activity of specific oxidases. The C_20_-GA-oxidases (i.e., GA20ox) and C_19_-GA-oxidases (i.e., GA3ox) act as rate-limiting enzymes within the last steps of the synthesis process, and their activity increases the pool of active GAs acting on intermediate or non-biologically active GAs. On the other hand, active GAs can be deactivated by other specific oxidases, mainly C_20_-GA-2-oxidases and C_19_-GA-2-oxidases (GA2ox). Balance between the activity of these different types of enzymes determines the GA content in plants, thus establishing these oxidases as the main targets for the GA regulation exerted by other compounds, genes, or phytohormones. However, a more detailed view of GA synthesis and homeostasis was recently reviewed [17].

The nuclear receptor GIBBERELLIN-INSENSITIVE DWARF1 (GID1) is responsible for the perception of GA. The GA–GID1 interaction enables the ubiquitination and degradation of DELLA proteins, which act as repressors of GA signaling. DELLA proteins belong to the GRAS family (based on the designation of GIBBERELLIC-ACID INSENSITIVE, [G]AI, REPRESSOR OF GA, [R]GA [A]ND SCARECROW, [S]CR), and the molecular mechanisms enabling them to block GA signaling have been already described [18]. Overall, a continuous balance between GA perception and DELLAs degradation governs the genetic responses to these phytohormones.

## 3. Vegetative Development

The ability of GA to control different aspects of plant development has driven a continuous effort to unravel the molecular mechanisms controlling these responses. In this section, we highlight relevant results recently achieved in GA research that have led to the identification of many genes and proteins in both model and non-model species. A detailed list of the identified genes in these and other processes and their activating or inhibiting role in GA signaling can be found in the Supplemental Tables (S1 and S2).

### 3.1. Shoot Elongation

**Figure 1:**
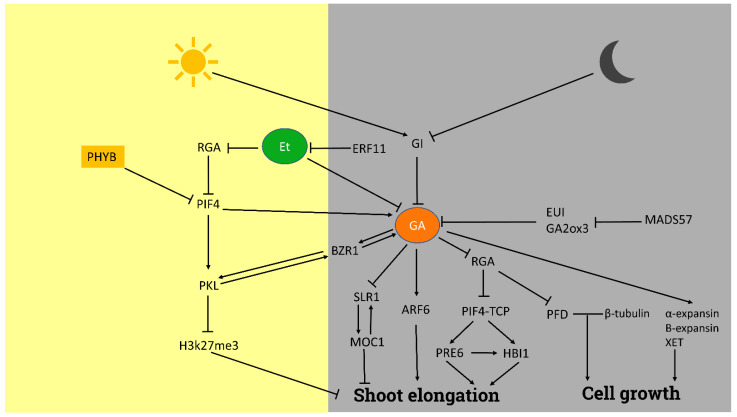
Schematic representation of the GA-related signaling involved in the process of shoot elongation. Yellow and grey background indicate light and dark conditions, respectively. Arrows indicate activation and blunt-end lines indicate repression or inhibition. See text for details and references. ARF6: auxin response factor 6, BZR1: brassinazole-resistant 1, ERF11: ethylene response factor 11, EUI: elongated uppermost internode, GA: gibberellins, GA2OX: gibberellin 2-oxidase, GI: gigantea, H3k27me3: 3 methylation of lysine 27 in histone 3, HBI1: homolog of bee2 interacting with ibh 1, MADS57: MADS box transcription factor 57, PFD: Prefoldins, PHYB: phytochrome B, RGA: repressor of GA, PIF4: phytochrome-interacting factor 4, PIF4-TCP: phytochrome-interacting factor 4-teosinte branched 1–cycloidea–pcf, PKL: pickle, PRE6: paclobutrazol resistance 6, SLR1: slender rice 1, XET: xyloglucan endotransglycosylase.

Shoot growth and development, major agronomical traits, are of great relevance for plant yield, architecture, and overall performance, and the activity of GA is believed to directly influence both processes (Figure 1). At the molecular level, GA favors plant elongation through cell growth regulation. According to the mechanisms elucidated in *Festuca arundinacea*, GA application promotes the transcription of xyloglucan endotransglycosylase (XET), α and β-expansins [19]. Besides, DELLA proteins physically interact with prefoldins and, after DELLA degradation induced by GA, free prefoldins are able to bind β-tubulins and stabilize them, thus affecting microtubules orientation and the direction of cell expansion [20]. Indeed, recent data suggest a close relationship between prefoldin activity and GA signaling. Expression analysis in the shoot apex of a prefoldin sextuple mutant of Arabidopsis showed the upregulation of the *GA2ox* gene in plants growing under short-day photoperiod. In contrast, the analysis revealed a reduced expression of PHYTOCHROME-INTERACTING FACTOR 4 (PIF4), a transcription factor (TF) closely related to GA responses (see below) and involved in the control of auxin-related genes [21]. Therefore, although more research is needed, there seems to be a close link between GA and prefoldins, that might help govern cell expansion and division. This results in the expansion of the cells in a GA-driven fashion.

The ability of GAs to control shoot elongation has been shown in agronomical relevant species such as rice. In this species, stem elongation and tiller number are regulated by the GA-induced degradation of SLENDER RICE 1 (SLR1), a DELLA protein that binds to tiller regulator MONOCULUM 1 (MOC1) preventing its degradation [22]. Furthermore, GA prevents interaction between SLR1 and KNOTTED1-LIKE HOMEBOX (KNOX) allowing panicle development [23]. Plant height, internode elongation, and panicle development are controlled by *OsMADS57*, a MADS-box gene that acts as a key regulator by repressing the expression of the cytochrome P450 monooxygenase *ELONGATED UPPERMOST INTERNODE* (*EUI*) and *OsGA2ox3* and, therefore, allowing GA accumulation [24]. It has been recently shown that a module comprising the F-box protein DWARF3 and the microRNA miR528 affects plant height in rice by modulating GA and Abscisic Acid (ABA) homeostasis [25]. In addition, the *ELONGATED INTERNODE* (*EI*) gene in tomato, which causes dwarfism, is related to the GA metabolic pathway as it encodes the *GA2ox7* gene, a catabolic enzyme within this pathway [26]. It has been shown that GA is also a key plant hormone controlling shoot elongation in carrot [2,27].

GA gradients are directly related to the elongation of roots and dark-grown hypocotyls in Arabidopsis, as seen with the use of a FRET-based biosensor [28]. As above mentioned, cooperative action of DELLA and light degrades PIF4 through the phytochrome B action, which is activated by ABA to prevent shoot growth [29]. However, under dark conditions and exogenous application of GA, PIF4 activates genes related to cell elongation [30]. Indeed, GA signaling and activity seem to depend on the plant circadian clock [31]. In line with this, DELLA proteins are stabilized during the daytime by GIGANTEA (GI), while GI degradation during nighttime allows GA activity. This finding suggests that GI is the key regulator of the circadian clock in hypocotyl elongation [32]. PIF3, PIF4, and PIF5 [30,33] activate several downstream genes that result in hypocotyl elongation, and at the same time lead to a positive feedback that drives the accumulation of GAs [28]. In addition to activating the brassinosteroid-related TF *BRASSINAZOLE RESISTANT 1* (*BZR1*) [34], GA induces PIF expression and degrades the RGA DELLA proteins, allowing the activity of the auxin-response factor ARF6. In dark conditions, recruitment of PICKLE (PKL) by PIF and BZR1 blocks the accumulation of H3K27me3 marks and permits the activation of growth-related genes [35]. Cellular growth and hypocotyl elongation are also promoted by PIF, BZR1, and ARF in Arabidopsis [36] but in an apparently independent manner [37]. Hypocotyl elongation in response to high temperatures has been found to be regulated through GA, at the posttranscriptional level by regulating the PIF4 activity in Arabidopsis [38] or GA_12_ transport from root to shoot in tomato, which seems relevant in the integration of day-night temperature oscillations [39]. *PIF4* activates *GA3ox* and interacts with TEOSINTE BRANCHED 1, CYCLOIDEA, PCF (TCP) TCP15/14, which enhances the expression of *GA20ox*, to promote hypocotyl elongation through *HOMOLOG OF BEE2 INTERACTING WITH IBH-1* (*HBI1*) and *PACLOBUTRAZOL RESISTANCE 6* (*PRE6*), among other genes. The role played by GA in the plant’s ability to adapt its growth patterns to changes in environmental temperature [40], suggests additional roles of GA in the response to outer cues.

An interaction of GA and ultraviolet-B light (UV-B) on shoot elongation has also been reported [41]. UV-B eases the cooperation between CONSTITUTIVELY PHOTOMORPHOGENIC 1 (COP1) and ELONGATED HYPOCOTYL 5 (HY5) to promote the accumulation of the RGA (DELLA) protein that leads to inhibition of hypocotyl elongation [42]. In addition, UV-B induces PIF4 and PIF5 degradation, thus reducing hypocotyl elongation [43]. These results show that light quality is also sensed and transduced in specific responses through GA-linked signaling routes.

The link between GA and light has also been shown to relate to the shade avoidance response. Under normal light conditions, CRYPTOCHROME 1 (CRY1) allows STENOFOLIA (STF) and DELLA accumulation and reduces GA levels in soybean and Arabidopsis [44,45]. However, in dark conditions CRY1 is inactive, allowing the GA-guided degradation of the DELLA proteins and the expression of genes activated by the released PIFs, which promote hypocotyl elongation [46]. GA also promotes shade-induced stem elongation as a manifestation of shade avoidance response in rice [47]. A shade-tolerant mutant of perennial ryegrass also supports the role of GA in this process, as it showed a reduced GA biosynthesis rate in the dark [48].

The complex framework of the GA effect on shoot elongation also involves the crosstalk of GAs with other phytohormones. The ETHYLENE-RESPONSIVE FACTOR 11 (ERF11) controls hypocotyl elongation by two independent but complementary pathways, repressing DELLA proteins and promoting the biosynthesis of GA, the latter via repression of ethylene biosynthesis genes [49]. In different species such as *Scirpus mucronatus* [50] and maize [51], ABA seems to inhibit shoot growth. In rice, strigolactones regulate shoot length by affecting GA homeostasis [52]. Other phytohormones, such as auxin and BRs, are involved in hypocotyl elongation through their interaction with GA [53].

Therefore, modulation of GA levels emerges as a key mechanism in the integration of outer (temperature, light quality) and inner cues (circadian clock) involved in shoot development, providing a connection with specific responses such as cell expansion and division.

### 3.2. Xylogenesis and Cellulose Production

The development of the secondary cell wall in plants as well as the formation of xylem, also referred to as secondary growth, are related processes that strongly influence growth and performance. Indeed, due to the direct relationship between xylogenesis and the production of wood and biomass, xylogenesis is an economically desirable process, particularly in trees. The proposed relationship of GA with cell and plant elongation also enables a role for these compounds in wood production, in order to help plants maintain large architectures. Accordingly, the proposed antagonistic relation of GA and ABA is also found in these processes, as the latter inhibits both shoot elongation and xylogenesis [54,55]. In recent years, it has been found that GA promotes cambial activity [56] and xylogenesis in trees [55,57]. Furthermore, the expression of three *CELLULOSE SYNTHASE* (*CESA*) genes in *Eucalyptus* (*CESA3*, *CESA4*, and *CESA7*), which are involved in xylem development, was induced by GA treatment [58]. Similarly, in birch, application of GA promoted xylem development and induced the expression of genes related to xylogenesis and cellulose production, such as *MYB*, *CESA*, and *PHENYLALANINE AMMONIA-LYASE* (*PAL*) [59]. Transgenic approaches showed an increase in secondary growth in plants overexpressing GA-related biosynthesis and signaling genes [60], while biomass production in poplar has been enhanced by the overexpression of the *GA20ox* gene [61]. In hybrid aspen, other wood traits, such as cell xylem length, have been shown to be positively modulated by GA [57,62]. Tian et al. (2016) [63] also reported modifications of wood properties in Populus by GA-responsive lncRNAs.

This xylogenesis-promoting effect of GA is not restricted to woody species as it has also been described in carrot [27], cotton [64], or celery [65]. Indeed, xylem proliferation is suppressed in tomato mutants with altered GA signaling [66]. The ability of GA to modulate xylem proliferation needs further exploration but, in the same manner as plant height control, it is a potential strategy to increase biomass production and optimize wood yield.

### 3.3. Root Development

Continuous root growth is essential for plants to explore the soil for nutrients and to provide physical support for the constant growth of the aerial parts. The three-dimensional structure of the root (root system architecture) drastically influences such functions. GA seems to have several relevant roles in root development from meristem development [67] to root nodulation for nitrogen fixation [68,69].

Historically, it was accepted that GAs were promoters of root growth. However, as it will be stated, the effect of GA on rooting responses is highly dependent on the species under study. The GA gradient between the apical division and the elongation zones in the root is strongly related to the fast growth of both roots and shoots in the dark [28]. The longitudinal GA gradient of growing roots is a result of differential GA biosynthesis and cellular permeability [70]. After germination, GA represses *RGA* and *ARABIDOPSIS RESPONSE REGULATOR 1* (*ARR1*). Stabilization of PIN transporter proteins due to the downregulation of *SHORT HYPOCOTYL 2* (*SHY2*), caused by the repression of *ARR1*, promotes elongation of the root [71]. On the other hand, ARR1 recruits DELLA proteins to reduce the GA effect and maintain the root meristem status [72]. Additionally, GA promotes root meristem cell divisions and enhances root growth [73], and GA is required for primary root growth in rice which is inhibited by GA deactivation [74]. Moreover, if GA supply from shoot to root stops, a biosynthesis feedback regulation mechanism is activated to ensure the optimal quantity of GA to maintain root growth [75]. Nevertheless, some evidence suggests that GA-induced root elongation produces thinner roots in carrot or *Pseudostellaria heterophylla* [27,76]. In sweet potato, whose agronomical profitability depends on its ability to transform adventitious roots in storage organs, the addition of GA promotes root lignification and reduces starch accumulation, thus preventing the shift from root to storage-root organ [77]. Similarly, in *Gladiolus hybridus*, GA prevents starch synthesis and corm development via *GhSUS2* activation [78]. On the other hand, in *Panax ginseng* and yam, GA promotes secondary growth of storage roots [79,80]. It has been suggested that a high ABA/GA ratio promotes tuber development, while GA preponderance delays tuber formation [81]. GA also promotes root growth by increasing the indole-3-acetic acid (IAA) content and reducing the synthesis of flavonols, which inhibit polar auxin transport [82]. Nevertheless, the relationship between GA and root growth is not always straightforward. There are many species in which they have minor effects [83], no effect, or even inhibitory effects [84]. However, the model discovered in *Arabidopsis* seems to prevail for most species. Therefore, it seems clear that GA affects root development and architecture beyond root length.

A promoting activity of GA on adventitious rooting has been suggested in oak and cherry [85,86]. Nonetheless, most studies on other species, such as tobacco [87], *Populus* [88], and *Pinus radiata* [89], point in the opposite direction. In hybrid aspen and Arabidopsis, GA inhibits adventitious rooting by affecting auxin transport [90]. The induction of adventitious roots depends on the ability of tissues to generate an auxin gradient, which relies on the auxin transport machinery. The inhibition of that transport by GA seems to underlie their inhibitory effect on this process. Adventitious rooting is inhibited in poplar transgenic plants overexpressing the histone deacetylase *PtHDT902*, which increases GA biosynthesis [91]. In line with this, adventitious rooting was improved when GA content was lowered by the overexpression of the *GA2ox* gene [92] or by the treatment with the GA biosynthesis inhibitor Paclobutrazol (PBZ) [93,94]. The analyses performed with the tomato GA mutant *procera*, which exhibits a loss of in vitro organogenic capacity to form shoots and roots, suggest that DELLA protein loss of function affects the cell-fate acquisition competence rather than the induction phase of the adventitious rooting process [95]. Interestingly, fruit development responses to exogenous auxin are enhanced in this mutant [96]. Therefore, results suggest that root developmental processes are affected by the crosstalk between GA and auxin. Indole-3-butyric acid (IBA) treatment inhibits GA synthesis in mung bean [97] and GA can have a negative effect on the adventitious rooting stimulated by IBA in apple tree [98]. It has been suggested that during adventitious root formation, the expression of *PIN* genes, which are auxin transporters, is repressed by GA [99]. The body of evidence for GA’s impact on adventitious rooting seems to indicate that this process is inhibited by GA in most species, although the effect seems to be species-specific. More research is needed to clarify the role of GA in rooting and their complex interaction with the auxin signaling and transport machinery.

In the case of lateral roots, results are also somehow contradictory. In *Populus*, the addition of GA promotes lateral root formation [88], but inhibits lateral root primordia initiation through interactions with auxin [100]. On the other hand, studies in rice revealed that the addition of exogenous GA_3_ reduces the number of lateral roots [92].

Therefore, GA seems to play a positive role in root growth and an inhibitory role in the development of adventitious roots, although the effect might be species specific and no unambiguous role can be attributed so far.

### 3.4. Other Vegetative Processes

Plants are able to react to physical interactions, and specific responses to contact stimuli are known as thigmomorphogenesis. Activation of the *GA2ox7* catabolic gene through mechanical contact leads to changes in plant morphology. This mechanism also enhances both biotic and abiotic stress resistance in plants, and the induced changes can be reversed by exogenous GA application, at least in Arabidopsis [101].

Meristems are a complex set of undifferentiated cells which by means of controlled divisions and positional cues give rise to new tissues. The shoot apical meristem (SAM) needs a low GA content to function properly. It has been reported that the KNOX-induced activity of *GA2ox* leads to the oxidative deactivation of GA allowing the normal functions of SAM [102]. The positive effect of GA in the axillary meristem formation has been reported in garlic [103]; however, a dual role of GA in bud break and bud dormancy has also been reported. In grapevine, GA synthesis leads to bud dormancy release [104], while in hybrid poplar bud break is allowed by the MADS12 TF induced downregulation of the *GA2ox* gene [105]. Thus, low GA content seems necessary for normal SAM function, while high levels induce the development of axillary buds. Moreover, modification of GA content and signaling affects tree branching, and hence can be used to modulate tree crown characteristics (reviewed in [60]). Therefore, adjustment of GA levels can be used to modify plant architecture, enabling their use for fine-tuned agronomic production.

Trichome emergence and formation are also modulated by GA [106,107]. Trichomes, protruded single epidermal cells, have many functions in plants, but are particularly relevant in stress responses. The activity of GA is also needed for cotton fiber elongation in ovule cultures, a particular type of trichomes [108]. Noteworthy, several feedback mechanisms ensure the fine-tuned control of GA on the formation of these organs. In Arabidopsis, GA promotes trichome development, a process controlled by *GLABROUS INFLORESCENCE STEM* (*GIS*), which may act upstream or downstream of SPINDLY (SPY), a negative regulator of GA signaling [109]. On the other hand, trichome formation is repressed by TEMPRANILLO (TEM), an inhibitor of GA biosynthesis genes and its related transporter NITRATE TRANSPORTER/NITRATE PEPTIDE FAMILY (NPF). TEM prevents the GA-induced expression of *GLABROUS 1* (*GL1*), *GL3*, *ENHANCER OF GLABRA3* (*EGL3*), and TRANSPARENT TESTA GLABROUS1 (TTG1) genes [110]. Moreover, the histone acetyltransferase 1 (HAT1) TF is a negative regulator of trichome initiation which acts through a negative feedback loop. GA induces the expression of *HAT1* that in turn represses GA biosynthesis [111]. In *Populus tomentosa*, GA acts coordinately with miR319a/TCP to control trichome formation, directly impacting defenses against herbivores [112]. Traditionally, it was assumed that GA has a negative role in the defense of plants against biotic stresses [17]. Once a plant is colonized by a pathogen, GA activity seems to worsen the effects. However, herbivory is a major biotic stress for plants and GA can help to avoid this stress through the induction of trichome formation.

Leaf senescence is a complex process regulated by many factors, including GA, with light and age of the plant playing a central role. The effect of GA in this process varies according to the species under study, suggesting an intricate relation with internal and external cues. In Arabidopsis, GA-induced degradation of DELLA allows the activity of NAC-LIKE ACTIVATED BY AP3/PI (NAP) leading to leaf senescence and chlorophyll degradation [113]. Foliar senescence is postponed by GA in a way apparently independent of light in *Alstromeria* [114]. In Chinese flowering cabbage, a rapidly senescing vegetable, leaf senescence is delayed due to the TCP-induced activation of GA biosynthesis [115], while exogenous GA treatment postpones the process by inhibiting the expression of the *BrWRKY6* TF [116]. In contrast, PIF4 interacts with the GA signaling pathway accelerating leaf senescence in maize [117] and Arabidopsis [118]. In Arabidopsis, early leaf senescence is also induced by the GA-driven activation of *SENESCENCE ASSOCIATED GENE* (*SAG*), *WRKY45* [119], and *WRKY75* [120] genes, in an age-related process. The divergence of results does not allow to unequivocally assert the direction of foliar senescence after GA application. More studies are necessary to elucidate the role of GA in this process.

## 4. Reproductive Development

**Figure 2:**
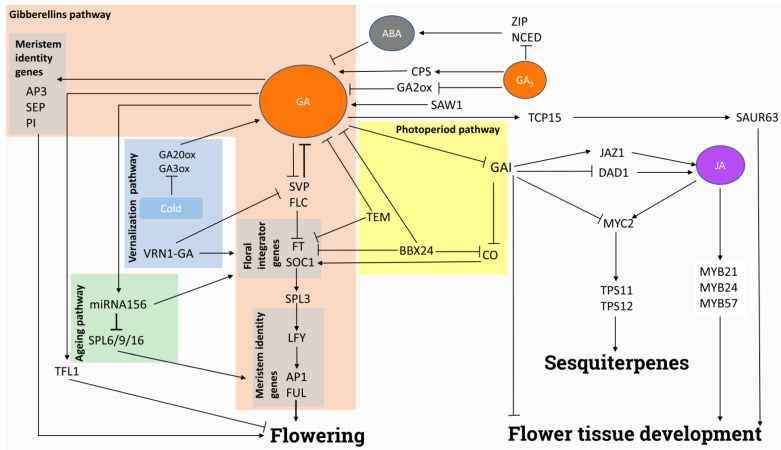
Schematic representation of the GA-related signaling involved in flowering and the development of the flower tissues. Arrows indicate activation and blunt-end lines indicate repression or inhibition. Orange box represents the GA flowering pathway, the blue box the vernalization flowering pathway, the yellow box the photoperiod flowering pathway and green box the aging flowering pathway. Grey boxes represent meristem identity genes and floral integrator genes. See text for details and references. ABA: abscisic acid, AP1/3: apetala 1/3, BBX24: B-box domain protein 24, CPS: *ent*-copalyl diphosphate synthase, CO: constans, DAD1: defective anther dehiscence 1, FLC: flowering locus C, FT: flowering locus T, FUL: fruitfull, GA2OX: GA2-oxidase, GA20OX: GA20-oxidase, GAI: gibberellic acid insensitive, JA: jasmonates, JAZ1: jasmonate zim domain, LFY: leafy, MYB: MYB domain protein, MYC2: transcription factor MYC2, NCED: nine-cis-epoxycarotenoid dioxygenase 2, SAUR63: auxin-responsive protein saur63, SAW1: swollen anther wall 1, SEP: sepallata, SLR1: slender rice 1, SPL: squamosa promoter binding protein-like, SOC1: suppressor of overexpression of CO1, SVP: short vegetative phase, TCP15: teosinte branched 1–cycloidea–pcf, TEM: tempranillo, TFL1: terminal flower 1, TPS11/12: terpene synthase 11/12, VRN1-GA: vernalization 1-GA, ZIP: HD-zipper family.

The relevance of GA in plant performance extends beyond their influence on growth and biomass yield. They also impact several reproductive-related processes, such as flowering and fruit formation. Due to the relevance of these processes for plant productivity, the related molecular mechanisms have been widely studied, particularly in crops, and many of the genes involved in these relevant traits have been outlined (Figure 2).

### 4.1. Flowering

In Arabidopsis, flowering is restrained by the joint action of several repressor proteins, including SHORT VEGETATIVE PHASE (SVP), FLOWERING LOCUS C (FLC), and Early Flowering 3 (ELF3). GA inhibits the expression of *SVP* and *ELF3*, through the action of the GAI ASSOCIATED FACTOR 1-TOPLESS RELATED (GAF1-TPR) complex, allowing the transcription of the floral integrator *SUPPRESSOR OF OVEREXPRESSION OF CONSTANS 1* (*SOC1*) and the leaf-derived mobile signal *FLOWERING LOCUS T* (*FT*) and the transition to the flowering process [121,122,123]. On the other hand, when days are too short, activation of SVP by PHOSPHORYLETHANOLAMINE CYTIDYLYLTRANSFERASE 1 (PECT1) reduces GA levels through the repression of *GA20ox* and inhibits floral transition of the shoot apex [124,125]. Under short-day conditions, GA activates *SQUAMOSA PROMOTER BINDING PROTEIN-LIKE 3* (*SPL3*) via *SOC1* and enhances the expression of *LEAFY* (*LFY*), which induces flowering by activation of *APETALA 1* (*AP1*) and *FRUITFULL* (*FUL*) [126,127]. In non-flowering promoting conditions, other genes of the *SPL* family have a key role in floral induction. This aging-dependent flowering pathway is mediated by miR156 in response to GA. SPL15 interacts with SOC1 to activate *FUL* and promote floral primordia development [128]. The indirect modulation of *SPL* genes by GA through miR156 has also been reported in Chinese chestnut [129]. On the other hand, under long-day conditions in Arabidopsis, DELLA prevents flowering by physically interacting with the photoperiod-related TF CONSTANS (CO), blocking its activity. Although GA signaling does not directly regulate CO levels, it mediates flowering by repressing the transcriptional activity of CO, the master regulator of the photoperiod flowering pathway, thus establishing a direct link between this route and GA [130]. Furthermore, histone deacetylation is required during the transition from vegetative growth in short days to flowering in long days which depends on photoperiod and intervenes in the GA signaling pathway [131]. In Chrysanthemum, flowering time and photoperiod are influenced by the *BBX24* gene, which inhibits the expression of the flowering promoters *CO*, *FT*, and *SOC1* genes as well as the GA biosynthesis genes *GA20ox* and *GA3ox* [132]. Similarly, in Arabidopsis, *TEM1* and *TEM2* genes link photoperiod and GA-dependent flowering of plants under short and long days by regulating the expression of genes (*GA3ox1* and *GA3ox2*) involved in GA biosynthesis [133].

Similar results have been found in other non-model species. *LFY* and *SOC1*, involved in floral meristem determinacy, are upregulated by GA in *Jatropha curcas* and chrysanthemums [134,135], although the induction seems more relevant in the latter. In *J. curcas*, the floral identity genes *AP3*, *PISTILLATA* (*PI*), and *SEPALLATA* (*SEP*) *SEP1-3* are activated by exogenous GA_3_ addition [134]. The application of exogenous GA_3_ to induce tree peony reflowering inhibits *GA2ox*, together with *ZIP* and *NCED*, and at the same time promotes the expression of *ent*-copalyl diphosphate synthase (*CPS*). Upregulation of *CPS* increases endogenous GA_3_ level and reduces ABA content, suggesting that changes in ABA/GA balance allow bud dormancy release and reflowering in autumn [136,137].

The relevance of GA extends to flowering regulation through the vernalization pathway [138]. *VERNALIZATION 1* (*VRN1*) gene promotes GA biosynthesis and favors the induction of flowering in Winter canola [139], Pak Choi [140], or cereal crops [141]. In addition, wheat spike development, via *SOC1*, is induced by GA under short days but only in presence of *VRN1* [142]. Moreover, GA plays a relevant role in the regulation of the flowering of cereal crops [141,143]. Thus, GA emerges as a potential target for flowering and crop yield modulation.

However, there seem to be some species-specific features concerning the flowering-promoting activity of GA. Indeed, the inhibitory effect of GA on flowering has been reported in several perennial trees [144] and in relevant tree crops such as sweet orange [145] and other citrus species [146]. In apple, the GA-induced repression of flowering takes place through the regulation of *TERMINAL FLOWER 1* (*TFL1*)-like genes [147]. In addition, GA repression by spermidine induces flowering in apple [148]. These contrasting effect of GA in some woody and non-woody species requires further research, but once again precludes the establishment of comprehensive patterns of GA responses.

### 4.2. Flower Formation and Fertilization

Several processes related to flower development are triggered by GA. In Arabidopsis, the GA-induced degradation of the DELLA proteins RGA, RGL1, and RGL2 promotes the formation of petal, stamen, and anther [149]. GAs also induce pollen formation as they allow cell wall development during meiotic cytokinesis [150]. Likewise, they stimulate filament elongation, by the activation of *TCP15*, which in turn induces *SMALL AUXIN UP RNA 63* (*SAUR63*) genes [151].

Despite their usual antagonistic effects, GA and JA act synergistically in several processes of flower development. In this context, GA induces the biosynthesis of jasmonates, which enhance the expression of several MYB TFs permitting the correct formation of stamen and development of male fertility [152]. Indeed, both the jasmonates-related repressor proteins JASMONATE ZIM-DOMAIN (JAZ) and DELLA act synergistically to repress filament development [153]. The *SWOLLEN ANTHER WALL 1* (*SAW1*) zinc-finger TF enhances *GA20ox3* expression in rice promoting microspore and anther development and dehiscence through *GAMYB*, while the same *GAMYB* gene is the target of miRNA159 in yellow lupin controlling pollen release [154,155]. In the presence of GA and jasmonates, *MYC2* induces *TERPENE SYNTHASE* genes *TPS11* and *TPS21*, permitting the emission of volatile sesquiterpenes necessary for flower development [156]. Therefore, GA-related induction of flower organ formation seems to rely on cooperation with jasmonates.

The ability of GA to regulate flowering is also linked to stress responses. At low temperatures, in rice, the drop of endogenous GA content due to the repression of the GA20ox3 and GA3ox1 genes and the increase in the SLR1 DELLA protein blocks pollen production and drives infertility, but the process can be reverted by adding exogenous GA [157]. Moreover, treatments with exogenous GA_3_ show that GA regulates anther and pollen development by improving cold tolerance in almond [158]. In rapeseed, male sterility is also produced after high-temperature treatments, which interferes with the GA signaling pathway [159]. Thus, the ability of GA to integrate temperature-linked information into the flowering process enables the adjustment of these responses under fluctuating conditions in the field.

Nonetheless, a negative effect of GAs in some flower formation processes has also been reported. They inhibit ovule primordia formation in both Arabidopsis and tomato, although the mechanism of this inhibition is different. In spite of acting synergistically in various developmental processes, BRs seem to downregulate *GA20ox1* during ovule formation in tomato, playing a negative role in GA biosynthesis during this process. In this way, BRs stabilize DELLA proteins allowing ovule primordia formation, but both these hormones act independently in Arabidopsis [160].

## 5. Fruit Development

The modulation of fruit traits by the application of phytohormones and plant growth regulators has the utmost relevance in agronomy and a significant economic impact. The molecular mechanisms by which GA influences such traits along with fruit formation are beginning to be understood, showing that the cross-talk of GA with other hormones such as ABA is key in fruit development [161] and ripening in species such as strawberry [162]. GA activates *ALCATRAZ* (*ALC*) genes which govern the fructification process in Arabidopsis [163]. In spite of being a key hormone in fruit set [164,165], GA prevents fruit ripening in strawberry [166], pear [165], or tomato through the inhibition of *RIPENING INHIBITOR* (*RIN*), *NON-RIPENING* (*NOR*), and *COLORLESS NON-RIPENING* (*CNR*) genes [167,168]. Besides, GA is involved in other fruit development-related processes. It promotes the expansion of cells, increasing fruit weight without any loss of quality traits in pineapple [169] and apple [170], control chlorophyll and carotenoid metabolism to produce orange regreening [171], and modulate tomato morphology and firmness by controlling the activity of Sly-miR159 [172] and *FIRM SKIN 1* (*FIS1*) [173], respectively.

The *SPATULA* (*SPT*) gene, as well as the DELLA proteins, repress fruit development in Arabidopsis. This process can be reverted by the addition of exogenous GA. However, *SPT* does not interact with GID1 and consequently, it cannot be degraded by GA. Nevertheless, it is suggested that *SPT* can interact with other unidentified TF which is degraded, in an example of a DELLA-independent GA-driven process [174].

The effect of GA on parthenocarpy has also been reported. It induces this process by controlling cell division and cell expansion in atemoya, whereas fruit set and parthenocarpy are induced in 2,4-D-treated pear plants through GA biosynthesis enhancement [175,176]. In line with this, the overexpression of the pear gene *PbGA20ox2* in tomato leads to parthenocarpic fruit formation [177].

These studies highlight the potential role of GA treatments in fruit growing since they seem to promote fructification and improvement of fruit quality in diverse species.

## 6. Seed Germination

**Figure 3:**
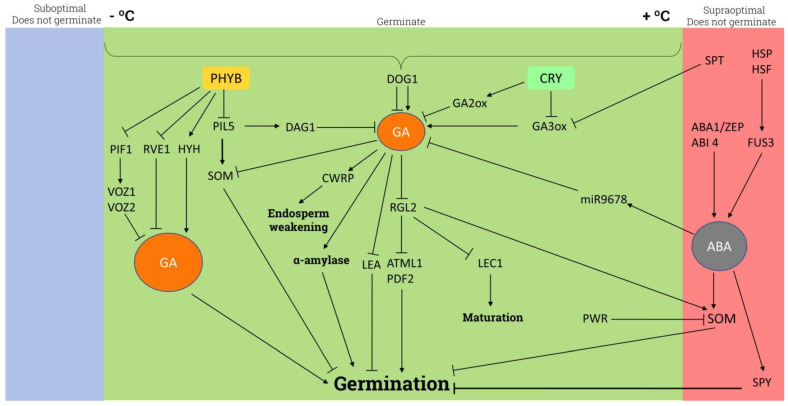
Schematic representation of the GA-related signaling involved in seed germination. Arrows indicate activation and blunt-end lines indicate repression or inhibition. See text for details and references. ABA1/ZEP: aba1/zeaxanthin epoxidase, ATML1: *Arabidopsis thaliana* meristem layer 1, CRY: cryptochrome, CWRP: cell-wall-remodeling-protein, DAG1: dof-type zinc finger DNA-binding protein, DOG1: delay of germination 1, FUS3: fusca3, GA2ox: GA2oxidase, GA3ox: GA3oxidase, HSF: heat shock factor, HSP: heat shock protein, HYH: hy5 homolog, LEA: late embryogenesis abundant, LEC1: leafy cotyledon 1, PDF2: protodermal factor 2, PHYB: phytochrome B, PIF1: phytochrome-interacting factor 4, PIL5: phytochrome-interacting factor-like 5, PWR: powerdress, RVE1: reveille 1, RGL2: rga-like protein 2, SOM: somnus, SPT: spatula, SPY: spindly, VOZ1/2: vascular plant one-zinc finger.

Determination of seed dormancy and the optimal moment to initiate germination is critical for improving plant livelihood and avoiding potential threats in the first stages of seedling development. It is well established that GA is the main hormone involved in seed dormancy breakdown, and the ABA/GA balance is the major regulator of seed dormancy and germination in wheat [178] and rice [179], with high GA content leading to decreased dormancy. In rice, the interplay between GA and BRs through the activity of *LEA* genes has also been shown to influence dormancy [180]. The main GA-related signaling pathways influencing seed germination are shown in Figure 3. GA, particularly GA_1_ and GA_4_, enhances seed germination in monocots such as macawn palm [181] and dicots such as red bayberry [182], while in Arabidopsis they promote the degradation of RGL2, the main repressor of seed germination [183]. DELLA degradation by the increase in GA in embedded Arabidopsis seeds also promotes seed germination by releasing ARABIDOPSIS THALIANA MERISTEM LAYER 1 (ATML1) and PROTODERMAL FACTOR2 (PDF2) from DELLA and allowing the expression of the *L1 box* gene [184]. In barley and rice, the α-amylase synthesis required during seed germination is induced by GAs [185,186,187].

*DELAY OF GERMINATION* (*DOG1*) gene expression and protein levels are directly correlated with seed dormancy, being a checkpoint for dormancy release in freshly harvested seeds in an ABA-independent fashion [188]. *DOG1* also regulates the temperature window for seed germination in different species. It modulates *GA20ox* expression in a temperature-dependent way, thus attaining the required levels of GA to induce *CELL-WALL-REMODELING PROTEINS* (*CWRP*) genes, which alter the mechanical properties of endosperm [189] allowing the weakening of endosperm in cress [190]. In addition, GA induces the enzymatic degradation of mannans in endosperm cell walls through the activity of endo-β-mannanase [191]. ABA-INSENSITIVE4 (ABI4) promotes ABA synthesis and *GA2ox7* expression, inhibiting GA biosynthesis and, therefore, maintaining seed dormancy and blocking germination [192,193]. In addition, ABA also represses germination through the activation of miR9678 reinforcing the importance of GA biosynthesis in the ABA/GA balance and the key role of this balance in wheat [194,195] or maize [196] seed germination. At low temperatures, GA deactivates *SOMNUS* (*SOM*) and promotes seed germination. However, at high temperatures, *SOM* expression is enhanced directly by DELLA proteins and ABA [197] and epigenetically by the AGAMOUS-LIKE 67-EARLY BOLTING IN SHORT DAYS (AGL67-EBS) complex [198], inhibiting GA synthesis and thus preventing seed germination. Moreover, at supra-optimal temperatures, HEAT SHOCK PROTEIN (HSP) and HEAT SHOCK FACTOR (HSF) activate FUSCA3 (FUS3) protein synthesis and accumulation. This process drives ABA synthesis and GA degradation, blocking seed germination [199]. *ABI3* and *SOM* activity is regulated, depending on temperature, by differential histone acetylation mediated by POWERDRESS (PWR), which represses *SOM* under normal temperature conditions [200]. These data show again the ability of GA to integrate temperature-linked information into gene expression.

Penfield et al. (2005) [201] showed that PHYTOCHROME-INTERACTING FACTOR3-LIKE5 (PIL5) and SPT are implicated in seed germination through the repression of *GA-3ox* gene and thus GA synthesis. In a light-dependent way, PIL5 inhibits seed germination. PIL represses GA biosynthesis genes and promotes GA catabolism in a direct and indirect way, activating *SOM* [202] and *DAG1*, which inhibits *GA3ox1* [203,204]. Furthermore, light conditions and GA control seed germination. In barley, red light has no effect on germination but blue light and cryptochrome activate *GA2ox* and inactive *GA3ox*, which reduces GA levels and blocks germination [205,206]. Nonetheless, in Arabidopsis, degradation of PIL5 by red light allows seed germination [207]. Upon light activation, phyB interacts with FAR RED ELONGATED HYPOCOTYL 3 (FHY3) and represses *REVEILLE 1* (*RVE1*) and *RVE2*, which promote seed dormancy by inhibiting GA biosynthesis and stabilizing RGL2 DELLA protein [208], and FHY3 enhances GA synthesis through *SPT* activation [209]. Seed germination is also repressed through the regulation of PIF target genes *VASCULAR PLANT ONE-ZINC FINGER* (*VOZ*) *VOZ1* and *VOZ2* zinc finger TFs mediated by the inhibition of GA synthesis. The active form of phyB represses *PIF1*, *VOZ1*, and *VOZ2* and enhances seed germination [210]. In addition, blue light also alleviates seed dormancy by phyB stimulation and phyA repression, and leads to GA synthesis and signaling through *HY5 HOMOLOG* (*HYH*) action in Arabidopsis [211], in a new example of light-quality integration driven by GA.

Environmental differences between wet and dry seasons modulate the germination of wolf apple seeds mainly due to GA and ABA seed content modification, allowing the germination only in the most favorable conditions [212]. Other factors such as maternal plant environment affect seed germination in Arabidopsis [213]. Recently, in soybean, Chen et al. (2020) [214] found that shading in mother plants promotes seed germination through GA biosynthesis enhancement and ABA synthesis repression.

H_2_O_2_ enhances seed germination in different species [215]. Reactive oxygen species (ROS), which induce ABA catabolism and GA biosynthesis genes, enhance seed germination and vice versa, ROS synthesis inhibition reduces significantly germination in wild cardoon [216]. On the other hand, 1-Cys Prx (*AtPER1*), a seed-specific anti-oxidant peroxiredoxin, is accumulated during seed development, maintaining seed dormancy through ROS inhibition [217]. Moreover, GA promotes embryo maturation by releasing LEAFY COTYLEDON 1 (LEC1) from DELLA interaction [218].

Additionally, GA seems to participate in somatic embryogenesis in *Medicago truncatula* [219] and Arabidopsis. Activation of FUS3 by the somatic embryogenesis-related TF AGAMOUS-Like 15 (AGL15) reduces the expression of the biosynthetic gene *GA3ox2* and induces the expression of *GA-2ox6*, involved in the inactivation of active GA, thus reducing GA content. Another embryogenesis-related gene, LEC2, is induced by GA and enhances the auxin-responsive genes *YUCCA* (*YUC*) *YUC2*, *YUC4*, and *INDOLE-ACETIC ACID-INDUCED PROTEIN 30* (*IAA30*), thus promoting somatic embryogenesis. In Arabidopsis, upregulation of *IAA30* expression along with low GA levels promotes somatic embryogenesis [220]. Thereby, the involvement of GA in this developmental process seems to rely on the interaction with the auxin signaling machinery.

## 7. Current and Potential Applications

The relevance of GA in modern agriculture cannot be underestimated. From the Green Revolution to nowadays crop production, manipulation of GA content has brought significant improvements in crop yield and quality.

Industrial production of the five classical phytohormones for agronomical or research purposes is an important business market worldwide that is led by Europe, which represents more than half of the market share, and followed by the USA (23.6%) and Asia (13.6%) [221]. In this market, the prominence of the USA stands out as the biggest supplier of these products in America and France, with the latter representing 21.9% of the European market share. However, it is expected that the countries of the Asia Pacific region, with a leading role for China, will exhibit significant growth opportunities for this market [222]. Concerning the data for each family of compounds, the market of phytohormone production was led in 2020 by cytokinins, which represent 38.1% of plant growth regulators market share, followed by auxins and GA [222]. However, this gap is expected to narrow in the coming years. The cytokinins market, mainly used for agricultural purposes, especially in cotton production, is expected to grow at a composed annual growth rate (CAGR) of 4.9% until 2027, reaching a market value of USD 2.4 billion, with the Asia Pacific region holding 41% of market share [223]. Auxins, which are the second largest plant growth regulators in terms of market share nowadays (dominated by North America with 38.7% of market share), are expected to grow at a CAGR of 4.5% until 2027 and a forecasted value of USD 1.1 billion. Auxin production growth will be driven by cotton production demanded by the textile sector and by auxin application for organic fruit and vegetable production [224]. Ethylene production is also expected to grow at a CAGR of 5.5%. Nevertheless, although it is expected that ethylene production will grow in all market segments, the main driver of this market, and its future evolution, is its use for plastic production and in the automotive industry [225]. The ABA market is projected to grow at a CAGR of 4.2% until 2028, which is expected to reach USD 0.65 billion thanks to investments in agriculture in Asia Pacific countries [226]. The global market linked to GA and related compounds was estimated at USD 500 million in 2015, although it is expected to grow up to USD 1.42 billion by 2027. A CAGR of 8.8% between 2017 and 2027 is forecasted, with a market growing at a 10% rate in the Asian Pacific zone followed by Europe, 8.6%, and North America, 8.1%, which is today the region leader in market share. The use of GA for fruit production, which represent 67.7% of its current market, is expected to grow at a rate of 8.7% in the forecasted period, followed by their use for the malting of barley, which is expected to grow at a rate of 10.7%, sugarcane yield and seed production [227]. Taken together, these reports indicate that the GA market is projected to witness the fastest growth rate among the five classical phytohormones, almost doubling the other ones. Distinct steps in the biosynthesis process of GA can be inhibited by the application of several compounds which are currently used in agriculture [14]. Greater knowledge of GA molecular activity routes and the identification of the key genes involved in GA responses could provide the tools needed for the improvement of the agronomic performance of crops, whether by means of genetic engineering or by the identification of cheaper compounds. In the present review, we have gathered the information available concerning the many aspects of plant biology in which GA plays a role, thus identifying new possible targets for crop improvement. With the world expected to attain 10 billion people by 2050, every possible advancement in agriculture is needed in order to feed the predicted population.

Among the several compounds applied in agriculture that influence GA levels, PBZ emerges as one of the more relevant as it not only reduces GA levels but also decreases ethylene production and increases cytokinin content. Although its use is under scrutiny and subject to tight regulations in some countries, and it has even been banned in other ones such as Sweden [228], PBZ is one of the most widely used GA inhibitors. It is expected that its use continues to grow in the coming years with a CAGR of 5%, reaching a global market value of USD 3.04 billion in the year 2028 from the current 2.06 [229]. Several agronomic traits are affected by PBZ treatment, including growth, water status, membrane stability, photosynthesis, etc. (reviewed in [230]). More detailed knowledge of the PBZ mode of action and the promotion of its application could provide an excellent manner to improve crop production and stress tolerance. Several other compounds have been found to inhibit GA synthesis in plants, and they are widely used in crop production [14]. However, we will focus on research that directly analyzes GA responses, and that might help in the development of novel strategies. As already mentioned, a better comprehension of GAs and their effects can lead to an increase in production or an improvement in quality traits. In forage crops such as *Medicago truncatula*GA is used to improve biomass production for land harnessing [231]. Shade avoidance response is a non-desirable trait in crops, but it may be modulated to increase wood production in Chinese red pine by means of specific GA treatment and light regimes [232]. Similarly, in hybrid poplar, the production of wood and biofuel can be increased by enhancing the expression of the GA biosynthesis gene *GA20ox* [233]. It is even possible to improve crops by ameliorating traditional practices such as grafting [234] or opening new markets by modifying the architecture and size of ornamental plants, such as orchids [235]. One of the most serious defects of cereal crops is the pre-harvest sprouting, which causes huge economic losses every year. It was found that in the *mir156* rice mutant the gene *IDEAL PLANT ARCHITECTURE 1* (*IPA1*) modulates multiple steps in the GA pathway, enhancing seed dormancy, representing an effective method to suppress pre-harvest sprouting in rice [236]. In tomato, GA treatments enhance seed germination, reduce sprouting time and, moreover, accelerate plant growth, being potentially useful to increase plant production and quality [237].

Environmental damage derived from modern agriculture practices has become a great concern in our society, and the ongoing climate change scenario could even worse the effects. A deep understanding of GA and its action mechanisms might help to solve this problem. Knowledge about DELLA proteins and nitrogen interactions could enhance grain yield in rice and reduce nitrogen fertilizers dependence via chromatin modulation [238]. GA biosynthesis alteration can be a new molecular target for weed population control, for reducing herbicide usage, and therefore for reducing environmental damage, as it has been shown in wild radish [239]. GAs are, as a general rule, seed germination promoters. Glyphosate, one of the most widely used herbicides in the world seems to inhibit the P450 cytochrome enzymes, interfering in the de novo synthesis of GA [240]. However, since GA reduced germination of the invasive *Heracleum sosnowskyi*, GA treatment emerges as a good method to control these species populations [241]. Christiaens et al. (2012) [242] reported that in pre-cooled *Helleborus niger* and *Helleborus x ericsmithii*, GA application induces early flowering and enhances the number and size of flowers. Similarly, in blueberry, the inflorescence number and vegetative growth increased with GA_3_ treatment [243]. However, in *Matthiola incana*, GA induced stem elongation but no effect on flowering was observed [244]. These results highlight another aspect of GA, as the species-specific responses and the variability found in some cases can discourage their application. As seen in the present review, these different responses can be significant between crop and forest species. A greater effort to unravel the reasons underlying these differences will increase confidence in GA application.

*Vitis* species are some of the most profitable crops worldwide, and GAs are extensively applied to improve their performance. GA addition increases grapes size [245], representing one of the first experiments carried out to use GA in agronomic production [246]. They are also used to reduce the density of bunches increasing fruit size [247,248] and decreasing bunch rot [249,250,251]. However, these results are not observed in all grapevine varieties [252]. Nowadays, the transcriptomics effects of GA are being studied to develop successful breeding and selection programs [253].

The positive effect on fruit yield has been reported in different species such as strawberry, pineapple, and blueberry [169,243,254]. Moreover, GA increases plant height [255] and grain yield in rice [256], maize, and soybean [257,258,259]. Based on these data, it has been suggested that the manipulation of the copy number of GA-related genes might be an interesting strategy to enhance plant yield [260]. On the other hand, early application of PBZ can increase potato yield, and its application is recommended in high-temperature zones [261].

Proper control of plant stress responses can avoid yield loss derived from adverse environmental conditions. GA has been found to modulate plant responses to stress. Usually, GA activity is repressed in the presence of different stresses to reduce growth and improve defense mechanisms [17]. Moreover, more specific research is needed to clarify the GA role in response to biotic stress. GAs can be used to fight biotic stress since they induce resistance to *Spodoptera frugiperda* [262] or to *Candidatus Liberibacter asiaticus* [263]. The role of GA on phytoremediation has also been shown, resulting in both ecological and economic benefits. When GA is combined with pressmud it allows sunflower plants to grow in chromium (Cr(VI)) contaminated soil by stabilization of Cr [264]. In addition, IAA and GA_3_ application to *Brassica juncea* enhance phytoremediation in soils contaminated with cadmium and uranium [265]. Foliar application of GA to *Corchorus capsularis* allows the phytoremediation of copper-contaminated soils [266], highlighting their putative role to improve crop production in polluted soils, a problem of increasing relevance worldwide.

As already seen, GA weighs in on a plethora of plant developmental processes. This knowledge could be used to promote its action, producing enriched functional foods [267]. By contrast, the inhibition of GA could be used to avoid GA-related proteins which can cause medical conditions such as allergic diseases to pepper, cedar [268], or strawberry [269]. Thus, a deeper knowledge of GA actions could be useful not only for scientific or productive purposes but also from a food safety point of view.

The development of precise gene-editing techniques has opened a new era in plant research, allowing for the accurate modification of gene sequences that can alter plant performance in a transgene-free manner. CRISPR/Cas9, the most popular of these techniques, is beginning to be applied in relevant crops with the aim of modifying GA-related responses to improve plant performance. For instance, signaling mutants in tomato have shown improved responses to water deficit conditions without lowering harvest index [66], while GA content modification through the modulation of GA20 oxidases has been reported in rice and maize [270,271,272]. As shown in the present review, many other GA-related target genes can be modified by these same means, thus opening the possibility for accurate and beneficial editing of crops and trees.

However, despite their paramount relevance, there might be alternatives to molecular manipulation and chemical agriculture. GA research started, as already seen, due to the study of a fungus that was able to synthesize this plant growth regulator. Since then, researchers have discovered many bacteria and fungi species that are part of the soil microbiome and that are able to produce gibberellins [273,274]. Thus, crop production can be improved through rhizosphere modification as has already been done [46,275].

## 8. Conclusions

Although historically shadowed in research by other phytohormones, the impact of GA in agriculture has driven an increased interest in its study. Nonetheless, the understanding of the molecular mechanisms involved in the GA-related responses still lags behind that of other phytohormones.

However, research in recent years has led to the identification of key genes involved in the responses to GA, thus enabling the design of more specific research and breeding programs. As shown above, careful planning is mandatory as several traits can be affected when modifying GA content or signaling routes. Nonetheless, we have shown that for some GA-related developmental processes, such as adventitious rooting or flowering, significant differences can be found between trees and crops, raising the question about the transferability of knowledge between species. Relevantly, GA seems to integrate specific cues into their responses, such as light quality or age of the plant, which might help explain those differences. Therefore, the present review might help researchers to plan their strategies by taking these differences into account. Besides, GA cooperation with other phytohormones in specific responses such as stress and flower formation is another significant issue that deserves greater attention. Moreover, temperature and light quality information seem to be integrated, at least in part, through the modulation of GA levels, thus offering new potential targets for improved plant performance through the use of biotechnological tools. In particular, the modulation of GA levels might be a potential tool for the development of plant varieties able to stand warmer temperatures and harsh conditions, thus assuring a more resilient vegetable production under a climate change scenario.

GA is a versatile plant growth regulator involved in a barrage of developmental processes. It is worth noticing their implication in xylogenesis, shoot elongation, root development, flowering, and seed germination. However, in most cases, GA activity seems to rely on its balance with ABA. Thus, future research should be focused not only on GA modulation itself but on its relationship with ABA, since the ABA/GA balance is a major modulator of physiological responses. The present review shows that, even if no direct link exists at the molecular level between ABA and GA on many occasions, both hormones act antagonistically in virtually every major physiological process, together influencing key plant development processes. Therefore, ABA content modulation, whether increasing its content through direct application of ABA or reducing its content with the use of specific inhibitors (abamine, abscinazole-E3M), represents a potential tool for the fine-tuning of crop responses.

The examples shown on the direct implications of GA in agronomical performance highlight the importance of these compounds in modern plant production and the potential applicability to other crop species, leading to a qualitative and quantitative improvement in agricultural production. As research advances, the role of GA in the Green Revolution might just be one of the many improvements for agriculture they could provide. Despite the research knowledge gathered so far, the greater benefits might just be yet to come.

## Supplementary Materials

The following are available online at https://www.mdpi.com/article/10.3390/plants11192650/s1, Table S1. Genes up-regulated or down-regulated by GA action in each physiological process and species reported in the review; Table S2. Genes or proteins reported in the review which activate or inhibit GA signaling or synthesis pathways in each developmental process.
