# Supplementary material for: Plant Development and Crop Yield: The Role of Gibberellins
Source: Plants (Basel). 2022 Oct 9;11(19):2650. doi: 10.3390/plants11192650 (PMC9571322; doi:10.3390/plants11192650)
Supplement: Supplementary file 1 [file plants-11-02650-s001.zip › plants-1925045-supplementary.pdf]

| Process | Host | Genes up-regulated | Genes down-regulated |
|---|---|---|---|
| Shoot elongation | *Arabidopsis* | ARF6 [35], BZR1[35], PKL[35], HBI1 [40], PRE6 [40] | RGA [35] |
| | *Festuca arundinacea* | XET [19], $\alpha$-expansin [19], β-expansin [19] | |
| | *Oryza sativa* | KNOX [23] | SLR1 [22], MOC1 [22] |
| Xylogenesis | *Eucalyptus* | CESA3 [58], CESA4 [58], CESA7 [58] | |
| | *Betula* | MYB [59], CESA [59], PAL [59] | |
| Root development | *Gladiolus hybridus* | SUS2 [78] | |
| | *Arabidopsis* | | RGA [71], ARR1 [71], SHY2 [71] |
| Adventitious rooting | *Malus sp.* | | PIN [99] |
| Trichome formation | *Arabidopsis* | GL1 [110], GL3 [110], EGL3 [110], TTG1 [110] | HAT1 [111] |
| Leaf senescence | *Arabidopsis* | NAP [113], SAG [119], WRKY45 [119], WRKY75 [120] | |
| | *Brassica rapa* | | WRKY6 [116] |
| Flowering | *Arabidopsis* | GAF1-TPR [121,276], SOC1 [121–123,127], FT [121–123], SPL3 [126,127], LFY [126,127], AP1 [126,127], FUL [126,127], CO [130] | SVP [121–123], ELF3 [123] |
| | *Jatropha curcas* | AP3 [134], PI [134], SEP1-3 [134], SOC1 [134], LFY [134] | |
| | *Malus sp* | TFL1 [147] | |
| | *Chrystantemum* | SOC1 [135], LFY [135] | |
| Flower formation and fertilization | *Arabidopsis* | RGA [149], RGL1 [149], RGL2 [149], TCP15 [151], SAUR63 [151], TPS11 [156], TPS21 [156], MYC2 [156], miR156 [124] | |

| | | | |
|---|---|---|---|
| | Chinese chestnut | miR156 [129] | |
| Fruit development | *Arabidopsis* | ALC [163] | SPT [174] |
| | *Solanum lycopersicum* | | RIN [167,168], NOR [167,168], CNR [167,168] |
| Seed germination | *Arabidopsis* | ATML1 [184], PDF2 [184]caca, L1 box [184], CWRP [189] | RGL2, SOM |
| | *Lepidium sativum* | CWRP [190] | |
| Embryo maturation | *Arabidopsis* | LEC1 [218] | |
| Somatic embryogenesis | *Arabidopsis* | LEC2 [220], YUC2 [220], YUC4 [220], IAA30 [220] | |

**Table S1**. Genes up-regulated or down-regulated by GA action in each physiological process and species reported in the review.

| **Process** | **Host** | **Host type** | **Gene/ Protein** | **GA signaling/synthesis pathway activation** | **GA singnaling/synthesis pathway inhibition** |
|---|---|---|---|---|---|
| Shoot elongation | *Oryza sativa* | Monocot | *EUI* [41] | X | |
| | | | PIF4 [42] | | X |
| | *Arabidopsis* | Dicot | GI [43] | X | |
| | | | PIF3 [42,44,45], PIF4 [42,44,45], PIF5 [42,44,45] | | X |
| | | | BZR1 [46] | | X |
| Adventitious rooting | *Populus* | Dicot | HDT902 [47] | | X |
| Bud break | Hybrid poplar | Dicot | MADS12 TF [48] | X | |
| Trichome formation | *Arabidopsis* | Dicot | TEM [11] | X | |
| | | | HAT1 [12] | X | |
| Leaf senescence | *Brassica rapa* | Dicot | TCP21 [49] | | X |
| Flowering | *Arabidopsis* | Dicot | SVP [30,50] | X | |
| | | | TEM [51] | X | |
| | *Chrysanthemum* | Dicot | BBX24 [52] | X | |
| | *Paeonia suffruticosa* | Dicot | CPS [53] | | X |
| | *Oryza sativa* | Monocot | SAW1 [54] | | X |

| | | | | | |
|---|---|---|---|---|---|
| Seed germination | *Arabidopsis* | Dicot | ABI4 [55,56] | X | |
| | | | SOM [57,58] | X | |
| | | | FUS3 [59] | X | |
| | | | PIL5 [60,61] | X | |
| | | | SPT [60,62] | X | X |
| | | | DAG1 [63,64] | X | |
| | | | FHY3 [62] | | X |
| | | | RVE [65] | X | |
| | | | HYH [66] | | X |
| | *Hodeum vulgare* | Monocot | CRY [67,68] | X | |
| Somatic embryogenesis | *Arabidopsis* | Dicot | FUS3 [40] | X | |

**Table S2**. Genes or proteins reported in the review which activate or inhibit GA signaling or synthesis pathways in each developmental process.
